# Enterolactone Reduces Telomerase Activity and The Level of Its Catalytic Subunit in Breast Cancer Cells

**DOI:** 10.22074/cellj.2017.4705

**Published:** 2017-05-17

**Authors:** Davod Ilbeigi, Mitra Nourbakhsh, Shahnaz Khaghani, Nahid Einollahi, Nejat Kheiripour, Zafar Gholinejad, Mohammad Alaee, Mostafa Saberian

**Affiliations:** 1 Department of Biochemistry, School of Medicine, Tehran University of Medical Sciences, Tehran, Iran; 2 Department of Biochemistry, School of Medicine, Iran University of Medical Sciences, Tehran, Iran; 3 Metabolic Disorders Research Center, Endocrinology and Metabolism Molecular, Cellular Sciences Institute, Tehran University of Medical Sciences, Tehran, Iran; 4 Department of Clinical Laboratory Sciences, Faculty of Allied Medical Sciences, Tehran University of Medical Sciences, Tehran, Iran

**Keywords:** Lignan, Enterolactone, Enterodiol, Telomerase, Breast Cancer

## Abstract

**Objective:**

There is a positive correlation between higher serum phytoestrogen concentrations and lower risk of breast cancer. The activation of telomerase is crucial for the growth of cancer cells; therefore, the aim of this study was to examine the effects of enterolactone (ENL) and enterodiol (END) on this enzyme.

**Materials and Methods:**

In this experimental study, we performed the viability assay to determine the effects of different concentrations of ENL and END on cell viability, and the effective concentrations of these two compounds on cell growth. We used western blot analysis to evaluate human telomerase reverse transcriptase catalytic subunit (hTERT) expression and polymerase chain reaction (PCR)-ELISA based on the telomeric repeat amplification protocol (TRAP) assay for telomerase activity.

**Results:**

Both ENL and END, at 100 μM concentrations, significantly (P<0.05) reduced cell viability. However, only the 100 μM concentration of ENL significantly (P<0.05) decreased hTERT protein levels and telomerase activity. Lower concentrations of ENL did not have any significant effects on telomerase activity and hTERT protein levels.

**Conclusion:**

High concentration of ENL decreased the viability of MCF-7 breast cancer cells and inhibited the expression and activity of telomerase in these cells. Although END could reduce breast cancer cell viability, it did not have any effect on telomerase expression and activity.

## Introduction

Breast cancer, one of the most common cancers in women, is an important public health problem with 1,384,155 estimated new cases worldwide and nearly 459,000 related deaths. The worldwide incidence of female breast cancer is estimated to reach approximately 3.2 million new cases per year by 2050 (1). Breast cancer incidence increases with age and more than 50% of patients are 65 years or older (2). Many factors contribute to the development and progression of breast cancer. Among them, estrogens play a crucial role, as high concentrations of circulating endogenous estrogens have been associated with increased breast cancer risk. Estrogens promote cell proliferation and tumor growth by binding to estrogen receptors (ERs) (3). Phytoestrogens are composed of various plant-derived compounds considered beneficial for human health. Phytoestrogens have a structure and function similar to estrogens. Due to structural similarity, phytoestrogens may compete with estrogens for binding to ERs. Although phytoestrogens may act as either weak estrogen agonists or antagonists, characteristically they are antagonists in pre-menopausal women and replace endogenous estrogens in post-menopausal women. Isoflavones, coumestans, and lignans are three major classes of phytoestrogens found in seeds, whole grains, berries, fruits, vegetables, and sprouts (4,5). 

Lignans are metabolized in the mammalian gut by bacteria to produce estrogenic enterolignans, such as enterolactone (ENL) and enterodiol (END), which are the major lignans present in serum, urine, bile, and seminal fluids (6). A reported inverse association exists between serum 17 β-estradiol (E2) and risk of breast cancer among premenopausal and postmenopausal women (7). ENL and END exert antiproliferative activities on breast cancer cells (8). However, the molecular mechanisms of the antiproliferative effects attributed to ENL and END are not entirely understood. 

Continuous proliferation of cancer cells depends on the presence of telomerase, an enzyme that builds the repetitive sequences of telomeres (9,10). The human telomerase reverse transcriptase catalytic subunit (hTERT) is responsible for telomerase activity. Its transcription is extremely regulated and only found in cells with high proliferative capacity. The level of hTERT mRNA expression is tightly correlated with telomerase activity in a variety of epithelial cancers that include cervical, breast, colon, ovarian, and renal carcinomas which emphasizes the importance of hTERT in cell proliferation (11). Breast cancer cells benefit from telomerase activity and its upregulation has been found in 92% of carcinoma in situ lesions and 94% of invasive breast cancers. Overexpression of hTERT in breast cancer cells and its absence from most normal tissues make telomerase an attractive target for diagnosis and therapy (12). In the current study, we examined the effects of ENL and END on telomerase activity in breast cancer cells. 

## Materials and Methods

This experimental study received approval from the Ethics Committee of Tehran University of Medical Sciences (91, 02, 30, 18016). 

### Cell culture

MCF-7 human breast cancer cells were purchased from Pasteur Institute of Iran. Cells were routinely maintained in RPMI 1640 supplemented with 10% fetal bovine serum (FBS, Gibco, UK), penicillin (100 U/ml), and streptomycin (100 µg/ ml) at 37˚C in a 5% CO_2_ incubator. All cell culture reagents were purchased from Gibco, UK. Prior to treatment, culture medium was exchanged with phenol red-free RPMI 1640 supplemented with 5% dextran-coated charcoal-stripped FBS. Treatment was performed with different concentrations of either ENL or END (Sigma, USA) dissolved in dimethyl sulfoxide (DMSO). Control cells were treated only with DMSO at concentrations less than 0.1%. 

### MTT assay

3-(4,5-dimethylthiazol-2-yl)-2,5-diphenyltetrazolium bromide (MTT, Sigma, USA) was used to determine the effect of different concentrations of ENL and END on cell viability and to find the optimum concentrations of these two compounds. Briefly, we seeded MCF-7 cells onto 96-well plates at a density of 2×10^4^ cells per well. After cell adhesion, they were treated with 0.001-100 µM concentrations, each, of ENL and END for 48 hours with medium being refreshed every 24 hours. Subsequently, the MTT solution was added and after 3 hours of incubation, we replaced the medium with 200 µl DMSO to dissolve the resultant formazan crystals. The absorbance of the resultant colored compound was measured at 570 nm in a microplate reader. Data were collected from three separate experiments and the percentage of ENL- and END-induced cell growth inhibition was determined by comparison to DMSO- treated control cells. 

### Western blot analysis

Protein expression of hTERT was examined by Western blot. MCF-7 cells were seeded in 25 cm^2^ flasks at a density of 1.5×10^6^ cells. Cells were treated for 48 hours with or without various concentrations of ENL and END (1, 10, 100 µM) and 10 nM of E2 as the positive control, then washed with phosphate-buffered saline (PBS) and lysed with RIPA lysis buffer that contained a mixture of protease inhibitors. Following quantification of total protein, 50 µg of the whole cell lysate was denatured in Laemmli buffer at 95˚C for 5 minutes, resolved on 8% sodium dodecyl sulfate-polyacrylamide gel electrophoresis (SDS-PAGE), and electrotransferred onto polyvinylidene fluoride (PVDF) membranes. The membranes were blocked in tris-buffered saline (TBS) that contained 0.05% Tween 20 and 3% bovine serum albumin (BSA) for 1 hour at room temperature. Blots were then incubated with rabbit anti-hTERT antibody (Novus Biologicals, USA) and rabbit anti-β-actin antibody (Cell Signaling Technology, USA) overnight at 4˚C. For visualization, blots were incubated with horseradish peroxidase (HRP)- conjugated secondary anti-rabbit antibody (Cell Signaling Technology, USA) for 1 hour. Membrane-bound secondary antibodies were visualized with a chemiluminescence HRP substrate (Roche Applied Sciences, Germany). Densitometry analysis was performed using ImageJ 1.17 (NIH, USA) to determine relative amounts of protein. 

### Telomerase activity assay

In order to investigate the effects of ENL and END on telomerase activity, we treated MCF- 7 cells with different concentrations of ENL and END (1, 10, 100 µM) and E2 (10 nM) for 48 hours. 

For quantitative analysis of telomerase activity, we used the telomeric repeat amplification protocol (TRAP) with the TeloTAGGG PCR ELISA PLUS kit (Roche Applied Sciences, Mannheim, Germany) according to the manufacturer’s instructions. In the TRAP protocol, the telomerase reaction product is amplified by polymerase chain reaction (PCR) (13). Briefly, samples were reacted with biotinylated synthetic primer and allowed to be elongated by telomerase. In a second step, these elongated products were amplified by PCR after which the PCR products were denatured and hybridized to a digoxigenin (DIG)-labeled, telomeric repeat-specific detection probe. The hybridization products were immobilized via the biotin-labeled primer to a streptavidin-coated microplate and detected by an anti-DIG antibody conjugated to peroxidase. Quantification was performed after addition of peroxidase substrate and color development, by measuring absorbance with a plate reader. 

### Statistical analysis

Statistical analyses were performed by one- way analysis of variance (ANOVA) using SPSS (version 20.0). Data were expressed as mean ± SD and P<0.05 were considered statistically significant. Each experiment was performed at least three times. 

## Results

### Enterolactone and enterodiol reduced MCF-7 cell viability

**Fig.1:**
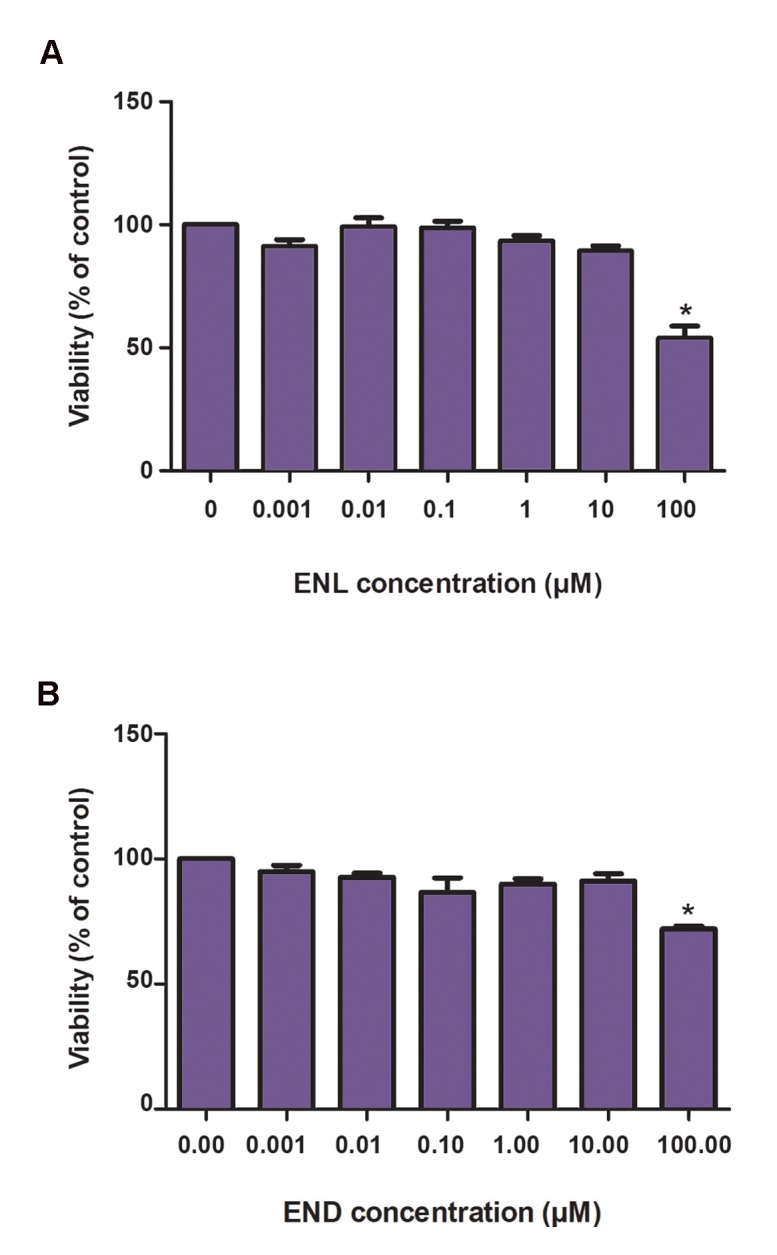
The effects of different concentrations of A. Enterolactone (ENL) and B. Enterodiol (END) on MCF-7 cell viability, determined by the MTT assay. Data are shown as percentage of control cells compared by one-way analysis of variance (ANOVA) and are the means of at least three separate experiments. *; Significant decrease compared to control P<0.05.

We performed the MTT assay to evaluate cell viability in response to ENL and END treatment. This test relies on the ability of viable cells to reduce tetrazolium salts and yield a colored compound readily quantified by a plate reader. Figure 1 shows that high concentrations (100 µM) of both ENL and END significantly decreased cell viability. Compared to control cells, we observed 58% of viable cells after treatment with END and 75% cells after treatment with END. This effect was more prominent for ENL. However, lower concentrations (0.001-100 µM) did not give rise to significant alterations in cell viability. 

### Enterolactone, but not enterodiol, decreased human telomerase reverse transcriptase catalytic

**Fig.2:**
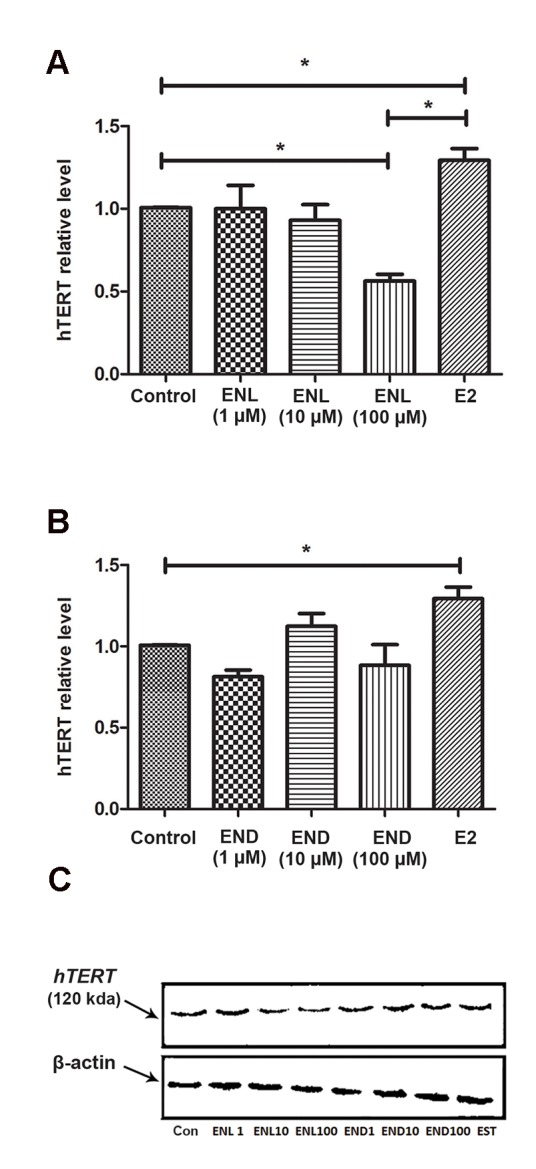
Enterolactone (ENL), but not enterodiol (END), decreased human telomerase reverse transcriptase catalytic subunit (hTERT) expression. Western blot analysis of hTERT protein levels in response to different concentrations of A. ENL and17β- estradiol (E2), and B. END and E2 for 48 hours. The values are the means of results of at least three separate experiments compared by one-way analysis of variance (ANOVA), and C. A representative western blotting of hTERT protein levels in MCF-7 cells after treatment with ENL, END, and E2. *; P<0.05.

subunit protein levels ENL treatment reduced hTERT protein levels. Figure 2A shows that the 100 µM concentration of ENL significantly (P<0.05) reduced protein levels of hTERT. Lower concentrations of ENL neither increased nor decreased hTERT levels. None of the concentrations of END could significantly alter hTERT protein levels (Fig.2B). In contrast, E2 significantly (P<0.05) increased protein levels of hTERT. 

### Enterolactone, but not enterodiol, repressed telomerase activity in MCF-7 cells 

**Fig.3:**
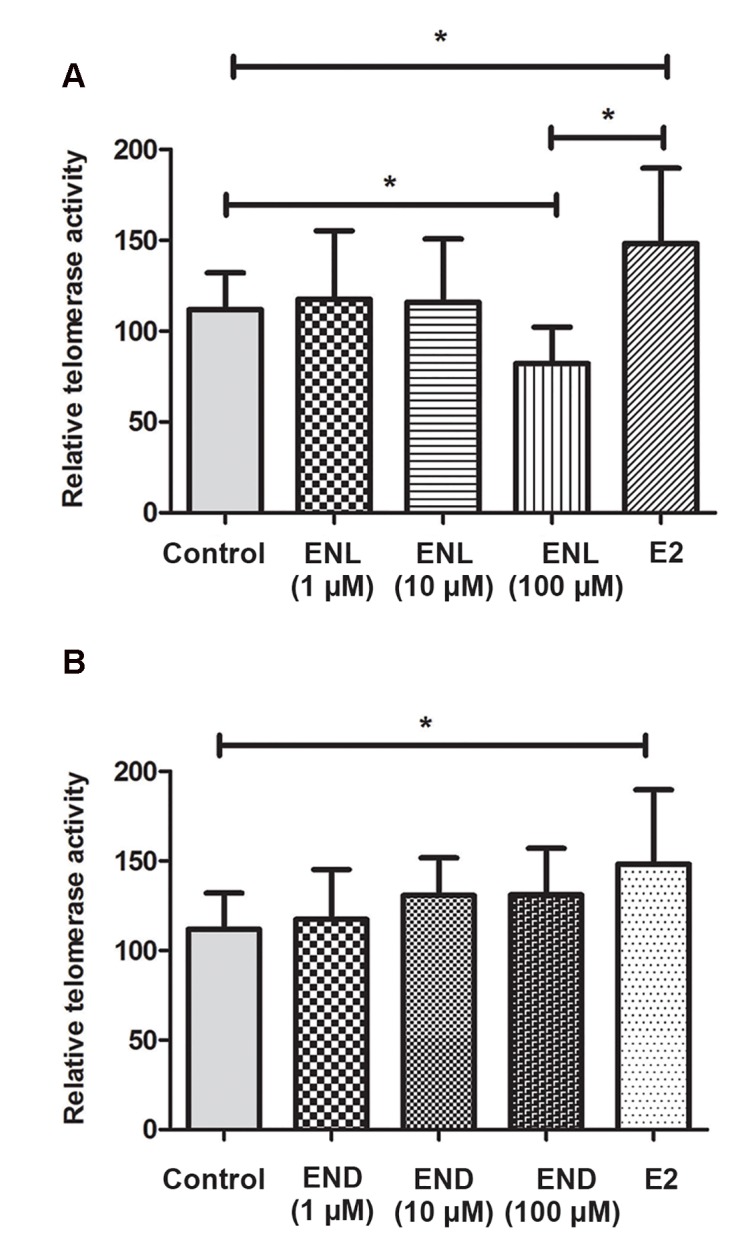
The effects of different concentrations of A. Enterolactone (ENL) and B. Enterodiol (END) on telomerase enzymatic activity in MCF-7 cells. Only the 100 µM concentration of ENL significantly reduced telomerase activity. Each value is the mean of three separate experiments performed in duplicates and compared by one-way analysis of variance (ANOVA). *; P<0.05.

As a consequence of the decline in hTERT protein levels, we expected to observe diminished telomerase activity after treatment with ENL. Figure 3 shows that treatment with the 100 μM concentration of ENL resulted in a significant reduction in telomerase activity in MCF-7 cells.Conversely, END did not have any significant effect on telomerase activity (Fig.3B). E2 increased telomerase activity in these cells, similar to its effect on hTERT protein expression. 

## Discussion

In this study, we have examined the effects of ENL and END on MCF-7 breast cancer cells which are ER positive and responsive to estrogens. Several epidemiologic and experimental studies indicate that a direct relationship exists between estrogen levels and the risk of breast cancer (3). Estrogens increase proliferation and viability of these cells, activate telomerase, and thus contribute to breast cancer progression (14). It is shown that phytoestrogens like genistein and ENL can bind ER and antagonize the effects of estrogen on cell growth and division (9,15,16). Therefore, unlike estrogens, phytoestrogens such as genistein and apigenin have been shown to retard cell growth and induce expression of the apoptotic proteins caspase-3 in prostate cancer cell line DU-145 and breast cell line MDA-MB-231 (17). 

High dietary intakes of plant lignans and increased exposure to enterolignans have been shown to be associated with reduced risks of ER- and progesterone receptor (PR)-positive postmenopausal breast cancer (5). Beneficial effects that include cell cycle arrest and induction of apoptosis were studied on several cancer cells, such as prostate and colon cell lines (18). ENL reduced proliferation and metabolic activity of prostate cancer cells (19). Here we showed that high concentrations of ENL and END decreased MCF-7 cell viability and thus might be useful for controlling the growth of breast cancer cells. The effects of ENL and END were previously investigated on the growth of MCF-7 xenografts in mice. In line with the current findings, ENL and END had the capability to significantly reduce tumor size (20). ENL inhibited the proliferative effect of E2 on MCF-7 breast cancer cells (9). 

A hallmark of cancer cells is their immortality which arises from high telomerase activity. Telomerase is able to restore telomeric repetitive sequences which protects the ends of chromosomes from degradation and are lost during cell division (21,22). hTERT, which is the catalytic and regulated subunit of telomerase, plays an essential role in the maintenance of telomeres and cell proliferation. Apoptosis is induced in response to downregulation of hTERT, independent to the telomerase enzymatic activity in human breast cancer cells (23). 

E2 can induce telomerase activity and cause overexpression of the hTERT catalytic subunit (24). Among phytoestrogens, the effects of genistein and daidzein on telomerase have been investigated and researchers reported their inhibitory effects on telomerase activity (25,27). Genistein has also been shown to prevent nuclear translocation of telomerase in other types of cancer such as head and neck, brain, and prostate (11,28,29). Many phytoestrogens stimulate growth at low concentrations and suppress growth at high concentrations, which suggests a biphasic effect on cell proliferation (30). Genistein shows its inhibitory effect on telomerase in pharmacologic concentrations (10-100 µM), while in lower concentrations it activates telomerase (11,31). Since plasma phytoestrogen concentrations mostly range from 100 nmol/l to 1 μmol/l (32), activation of telomerase by genistein may be expected rather than its inhibition. The reported median serum E^2^ concentration is 13.8 nmol/L (range: 0-95.6 nmol/L) in men and 16.6 nmol/L (range: 0-182.6 nmol/L) in women (33); the serum concentration of ENL can reach the maximum amount of 385 nmol/L after 6-week supplementation with lignans (34). Thus, low concentrations of ENL are mostly found in the circulation. 

The effect of lignans on telomerase has not been previously studied. In this study, we showed for the first time that ENL, but not END, decreased both telomerase activity and its protein levels. This inhibition has occurred with a high dose of ENL, whereas low doses had no effect on telomerase activity or expression. In contrast to genistein, ENL does not have a biphasic effect and this makes ENL a safer phytoestrogen compared to genistein. Interestingly, although both ENL and END reduced cell viability, only ENL inhibited telomerase activity which demonstrated that a specific mechanism exists for this effect. 

ER inhibition is an important approach in the treatment of ER-dependent cancers. Estrogenic compounds can interact with ER and modulate its function. Therefore, they are candidates for cancer management (35). ENL is able to affect ER signaling and modulate attachment of ER to estrogen response element (ERE) (36). The hTERT promoter contains an ERE (37). On the other hand, phytoestrogens can exert rapid non- genomic functions. For example, activation of AKT and focal adhesion kinase (FAK) is reported for genistein, daidzein, and resveratrol (35,38). Interestingly, activation of AKT pathway leads to hTERT induction (38). Whether the effect of ENL on telomerase is mediated by ER or non-genomic signaling pathways requires further investigations. ENL shows antiproliferative properties and apoptosis induction in colon cancer cells which implies non-estrogenic functions for ENL and suggests a benefit for oral consumption of ENL (39,40). 

The exact mechanism of the effect of ENL on telomerase requires additional investigations. It has been suggested that changes in the expression of the DNA licensing genes, increased expression of the PTEN tumor suppressor gene, or alterations in some microRNAs may be responsible for the antiproliferative activity of ENL (41). Similar mechanisms may be involved in the effect of ENL on telomerase; however their elucidation needs further studies. 

## Conclusion

We have provided evidence for diminished cell viability by ENL and its inhibitory effects on telomerase in a breast cancer cell line which suggest a benefit for ENL in breast cancer management. 
